# Region-Related Differences in Short-Term Synaptic Plasticity and Synaptotagmin-7 in the Male and Female Hippocampus of a Rat Model of Fragile X Syndrome

**DOI:** 10.3390/ijms25136975

**Published:** 2024-06-26

**Authors:** Giota Tsotsokou, Athina Miliou, George Trompoukis, Leonidas J. Leontiadis, Costas Papatheodoropoulos

**Affiliations:** Lab of Physiology-Neurophysiology, Department of Medicine, University of Patras, 265 04 Patras, Greece; panagiotatsotsokos@gmail.com (G.T.); athinamhliou@gmail.com (A.M.); geortrobjs@gmail.com (G.T.); ljleontas@gmail.com (L.J.L.)

**Keywords:** synaptotagmin-7, hippocampus, fragile X syndrome, *Fmr1*, short-term synaptic plasticity, synaptic transmission, septotemporal, dorsoventral, sex, rat

## Abstract

Fragile X syndrome (FXS) is an intellectual developmental disorder characterized, inter alia, by deficits in the short-term processing of neural information, such as sensory processing and working memory. The primary cause of FXS is the loss of fragile X messenger ribonucleoprotein (FMRP), which is profoundly involved in synaptic function and plasticity. Short-term synaptic plasticity (STSP) may play important roles in functions that are affected by FXS. Recent evidence points to the crucial involvement of the presynaptic calcium sensor synaptotagmin-7 (Syt-7) in STSP. However, how the loss of FMRP affects STSP and Syt-7 have been insufficiently studied. Furthermore, males and females are affected differently by FXS, but the underlying mechanisms remain elusive. The aim of the present study was to investigate possible changes in STSP and the expression of Syt-7 in the dorsal (DH) and ventral (VH) hippocampus of adult males and females in a *Fmr1*-knockout (KO) rat model of FXS. We found that the paired-pulse ratio (PPR) and frequency facilitation/depression (FF/D), two forms of STSP, as well as the expression of Syt-7, are normal in adult KO males, but the PPR is increased in the ventral hippocampus of KO females (6.4 ± 3.7 vs. 18.3 ± 4.2 at 25 ms in wild type (WT) and KO, respectively). Furthermore, we found no gender-related differences, but did find robust region-dependent difference in the STSP (e.g., the PPR at 50 ms: 50.0 ± 5.5 vs. 17.6 ± 2.9 in DH and VH of WT male rats; 53.1 ± 3.6 vs. 19.3 ± 4.6 in DH and VH of WT female rats; 48.1 ± 2.3 vs. 19.1 ± 3.3 in DH and VH of KO male rats; and 51.2 ± 3.3 vs. 24.7 ± 4.3 in DH and VH of KO female rats). AMPA receptors are similarly expressed in the two hippocampal segments of the two genotypes and in both genders. Also, basal excitatory synaptic transmission is higher in males compared to females. Interestingly, we found more than a twofold higher level of Syt-7, not synaptotagmin-1, in the dorsal compared to the ventral hippocampus in the males of both genotypes (0.43 ± 0.1 vs. 0.16 ± 0.02 in DH and VH of WT male rats, and 0.6 ± 0.13 vs. 0.23 ± 0.04 in DH and VH of KO male rats) and in the WT females (0.97 ± 0.23 vs. 0.31 ± 0.09 in DH and VH). These results point to the susceptibility of the female ventral hippocampus to FMRP loss. Importantly, the different levels of Syt-7, which parallel the higher score of the dorsal vs. ventral hippocampus on synaptic facilitation, suggest that Syt-7 may play a pivotal role in defining the striking differences in STSP along the long axis of the hippocampus.

## 1. Introduction

Fragile X syndrome (FXS) is a neurodevelopmental disorder and one of the most common inherited forms of intellectual disability [1,2,3,4], often associated with autistic behaviors [3,4,5]. FXS is primarily caused by a mutation leading to a transcriptional silencing of the *Fmr*1 gene [6,7,8], and to an insufficient expression or total loss of fragile X messenger ribonucleoprotein (FMRP), which is broadly expressed throughout the body, displaying high levels in the brain [9,10]. Behaviorally, FXS is characterized by deficiencies in social communication, learning and memory, hypersensitivity, anxiety, and seizures [2,11].

The main actions of FMRP are found in the synapses [7,12,13,14,15,16,17,18], and the numerous mRNA targets of FMRP are localized in the neuronal dendrites, where it modulates local translation of several synaptic proteins, including the glutamate receptor subunits [19,20] that are involved in long-term synaptic plasticity and memory formation [21,22]. Furthermore, FMRP can directly modulate other proteins, such as ion channels [23,24], while the loss of FMRP in FXS leads to increased levels of several proteins in the brain [13,14,25,26].

A great deal of the previous research concerning the consequences of FMRP loss on neuronal function has been focused on the postsynaptic compartment, and it is generally thought that most of FMRP’s effects are related to synaptic plasticity [13,27]. The loss of FMRP is also associated with the disrupted short-term processing of neural information, including sensory processing and working memory [28,29]; these forms of neural information processing are proposed as engaging with short-term forms of activity-dependent synaptic plasticity [30,31], which are thought to be critically supported by the Ca^2+^-dependent mechanisms that engage with the components of the neurotransmitter release machinery [32]. Interestingly, FMRP is expressed presynaptically [17,18,33,34], and recent evidence has revealed the significant actions of FMRP in modulating presynaptic function [12], suggesting its involvement in the presynaptic forms of synaptic plasticity. For instance, the targets of FMRP include the mRNAs that encode a plethora of presynaptic proteins involved in the process of neurotransmitter release [19,35]. For example, FMRP regulates neurotransmitter release by modulating calcium-dependent potassium channels [24] and voltage-gated calcium channels [36]. Also, FMRP contributes to sustaining high-frequency synaptic activity by enhancing vesicle recycling through activity-dependent bulk endocytosis [37]. It is worth noting that some of these presynaptic actions are translation independent [24].

Synaptic facilitation, a form of short-term synaptic plasticity (STSP) that is manifested as an increase in the synaptic response upon the rapidly repetitive activation of the presynaptic terminal, is attributed to a transient increase in the transmitter release probability resulting from the accumulating Ca^2+^ in the presynaptic terminal during repetitive activation [32,38]. Thus, STSP may be a result of the modulation of the neurotransmitter release process, which is fundamentally supported by several presynaptic proteins, including synaptotagmins, synaptobrevin, syntaxin, and SNAP-25 [39]. Synaptotagmins constitute a protein family of 17 members that function as calcium sensors [40,41]. Notably, synaptotagmin-1 (Syt-1) displays low Ca^2+^ affinity and is needed for synchronous neurotransmitter release [42,43], while synaptotagmin-7 (Syt-7) displays high calcium affinity and functions as a Ca^2+^ sensor that triggers synaptic vesicle fusion with the plasma membrane for conditions with low Ca^2+^ concentrations [44,45,46]. The experimental data suggest that Syt-7 plays a significant role in the asynchronous neurotransmitter release that occurs during repetitive synaptic activation [42,47], thereby significantly contributing to synaptic facilitation [48,49,50,51,52] and normal brain function [53,54]. Notably, Syt-7 may facilitate neurotransmitter release during the period that follows an action potential reaching a presynaptic terminal, by effectively sensing the low levels of Ca^2+^ during this period [55]. Interestingly, a previous report suggested that Syt-1 and Syt-7 may be modulated by FMRP [19]. However, whether the suppressed expression of FMRP affects the levels of synaptotagmins in the brain is still not known. We hypothesized that the loss of FMRP in FXS may alter STSP by affecting the expression and/or the function of Syt-7. Although changes in STSP have been found in immature FXS animals [15,17,24,56,57], the effects of FXS on STSP in adult animals have not yet been sufficiently clarified [58].

The dorsal (DH) and ventral (VH) hippocampus express remarkably different patterns of STSP [59,60,61,62,63,64,65,66,67,68,69,70,71], and have recently been shown to respond differently to FXS with respect to network excitability, GABAergic inhibition, and rhythmogenesis [72,73]. It is, therefore, necessary to separately study the possible effects of FXS on STSP in the two segments of the hippocampus. Also, it is essential to understand the distinct FXS-associated behavioral disturbances encountered in the two sexes [74,75], especially in view of the current under investigation of females with FXS/autism disorder [76,77].

Here, we show that two forms of STSP, namely the paired-pulse ratio (PPR) and frequency facilitation/depression (FF/D), as well as the protein expression of Syt-1 and Syt-7, are not altered in the hippocampus of adult males with FXS. However, the PPR is increased in the ventral hippocampus of adult female littermates. Furthermore, except in *Fmr1*-knockout (KO) females, the DH displays higher levels of Syt-7 compared to the VH, which parallels the difference in STSP between the two segments of the hippocampus.

## 2. Results

### 2.1. Genotype Affects Synaptic Facilitation in VH of Female Rats

**Figure 1:**
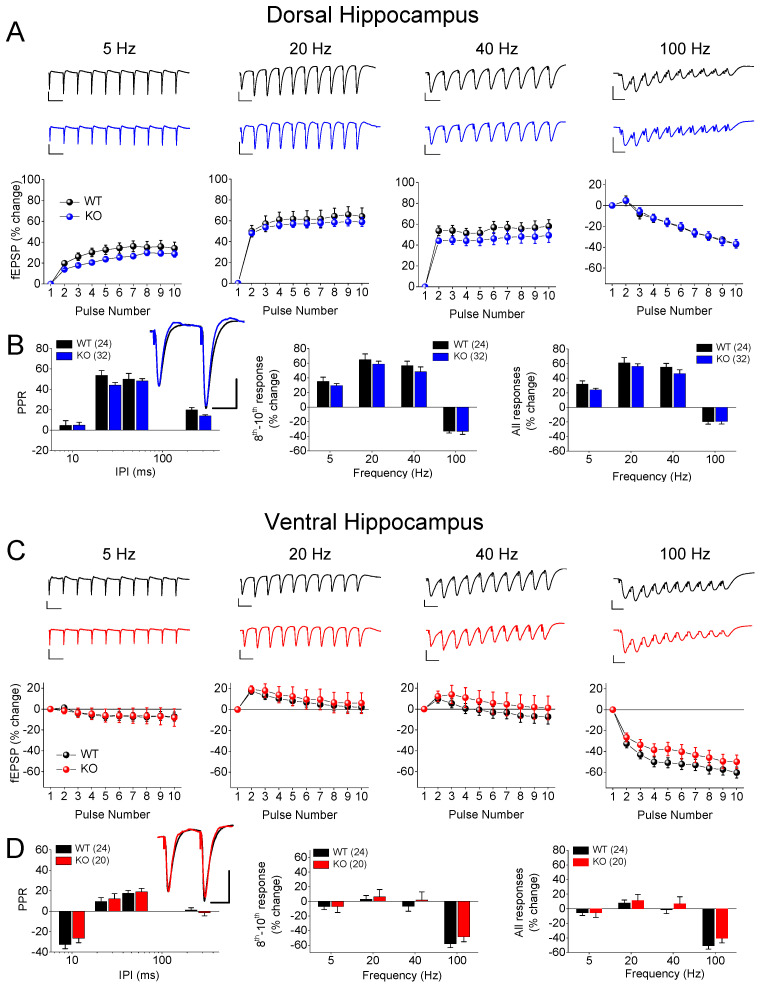
Genotype does not affect short-term synaptic plasticity (STSP) in adult male dorsal hippocampus (DH) or ventral hippocampus (VH). Results are shown for DH (**A**,**B**) and VH (**C**,**D**). (**A**,**C**) Representative traces of synaptic responses at 5 Hz, 20 Hz, 40 Hz, and 100 Hz stimulation trains (**top panels**), and corresponding percent change in responses during stimulation trains (**bottom panels**) are shown for wild-type (WT) and *Fmr1*-knockout (KO) male rats. (**B**,**D**) Average paired-pulse ratio (PPR) at four different inter-pulse intervals (IPIs) (**left**), percentage of steady-state response (average of 8th-10th responses) plotted as function of stimulus frequency (**middle**), and percentage of all conditioned responses (average of 2th–10th responses) plotted as function of stimulus frequency (**right**). Superimposed sample traces of paired-pulse responses from WT and KO are shown in inserts; scale bars: 25 ms, 1 mV. Numbers in parentheses indicate number of hippocampal slices used in analysis.

**Figure 2:**
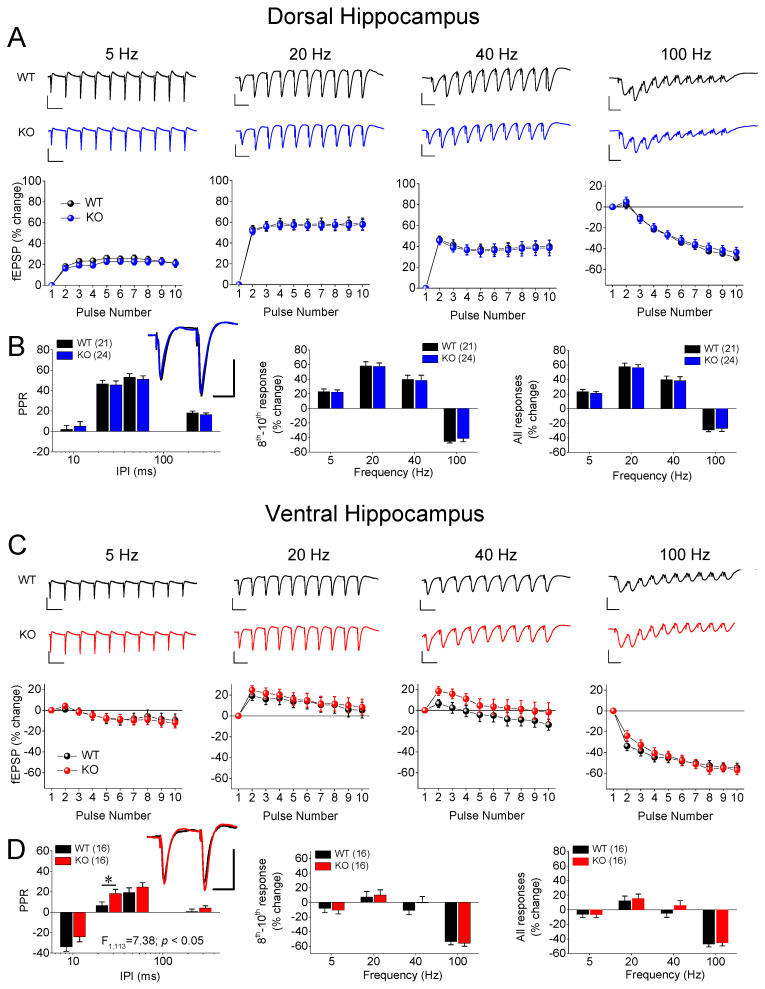
Genotype affects short-term synaptic plasticity (STSP) in adult female ventral hippocampus (VH) but not dorsal hippocampus (DH). Results are shown for DH (**A**,**B**) and VH (**C**,**D**). (**A**,**C**) Representative traces of synaptic responses at 5 Hz, 20 Hz, 40 Hz, and 100 Hz stimulation trains (**top panels**), and corresponding percent change in responses during stimulation trains (**bottom panels**) are shown for wild-type (WT) and *Fmr1*-knockout (KO) female rats. (**B**,**D**) Average paired-pulse ratio (PPR) at four different inter-pulse intervals (IPIs) (**left**), percentage of steady-state response (average of 8th–10th responses) plotted as function of stimulus frequency (**middle**), and percentage of all conditioned responses (average of 2th–10th responses) plotted as function of stimulus frequency (**right**). Superimposed sample traces of paired-pulse responses from WT and KO are shown in inserts; scale bars: 25 ms, 1 mV. Numbers in parentheses indicate number of hippocampal slices used in analysis. Asterisk (*) in (**D**) denotes statistically significant difference between WT and KO at *p* < 0.05 (independent *t*-test).

We examined two phenomena of STSP using a frequency stimulation protocol. Specifically, we studied the PPR and FF/D of the synaptic responses in the DH and VH of wild-type (WT) and KO male and female rats. A frequency stimulation was applied using similar conditioning responses (fEPSP, mV/ms) in the WT (0.68 ± 0.01) and KO (0.71 ± 0.01), and in the DH (0.69 ± 0.01) and VH (0.69 ± 0.011). We found that both the DH and VH of the male rats exhibited a similar pattern of STSP for the two genotypes (Figure 1), as the PPR, frequency facilitation, and frequency depression did not significantly differ between the WT and KO male rats in either the DH (Figure 1A,B) or VH (Figure 1C,D). Specifically, we found no statistically significant effect of genotype on the PPR of the DH (WT = 24 and KO = 32, *F*_1,205_ = 3.41, *p* = 0.066) or VH (WT = 24 and KO = 20, *F*_1,162_ = 0.54, *p* = 0.46), or on the FF/D of the DH (WT = 24 and KO = 32, *F*_1,205_ = 1.93, *p* = 0.167; and *F*_1,205_ = 2.7, *p* = 0.102; for the steady-state responses and all conditioned responses, respectively) or VH (WT = 24 and KO = 20, *F*_1,205_ = 1.14, *p* = 0.29; and *F*_1,205_ = 1.63, *p* = 0.20; for the steady-state responses and all conditioned responses, respectively). In contrast, we found a significant effect of genotype on the STSP of the VH, but not the DH, of the female rats (Figure 2). More specifically, we found that either the PPR (WT = 21 and KO = 24, *F*_1,164_ = 0.02, *p* = 0.89) or FF/D (WT = 21 and KO = 24, *F*_1,164_ = 0.01, *p* = 0.93; and *F*_1,164_ = 0.06, *p* = 0.81; for the steady-state responses and all conditioned responses, respectively) did not significantly differ between the WT and KO female DH (Figure 2A,B). However, we observed a significantly higher PPR at the inter-pulse interval (IPI) of 25 ms, but at no other IPIs, in the VH-KO compared to the VH-WT (IPI = 25 ms, independent *t*-test, t_28_ = −2.14, *p* = 0.041; all IPIs, ANOVA, *F*_1,113_ = 7.4, *p* = 0.008) (Figure 2C,D). Furthermore, as in the DH, the genotype did not significantly affect the FF/D in the VH of the female rats (WT = 21 and KO = 24, *F*_1,113_ = 0.24, *p* = 0.63; and *F*_1,113_ = 0.98, *p* = 0.32; for the steady-state responses and all conditioned responses, respectively). These results suggest that *Fmr1*-KO is associated with frequency-dependent alterations in the properties of STSP, specifically in the VH of female, but not male, rats.

### 2.2. STSP Differs between DH and VH from Male and Female Rats of Both Genotypes

**Figure 3:**
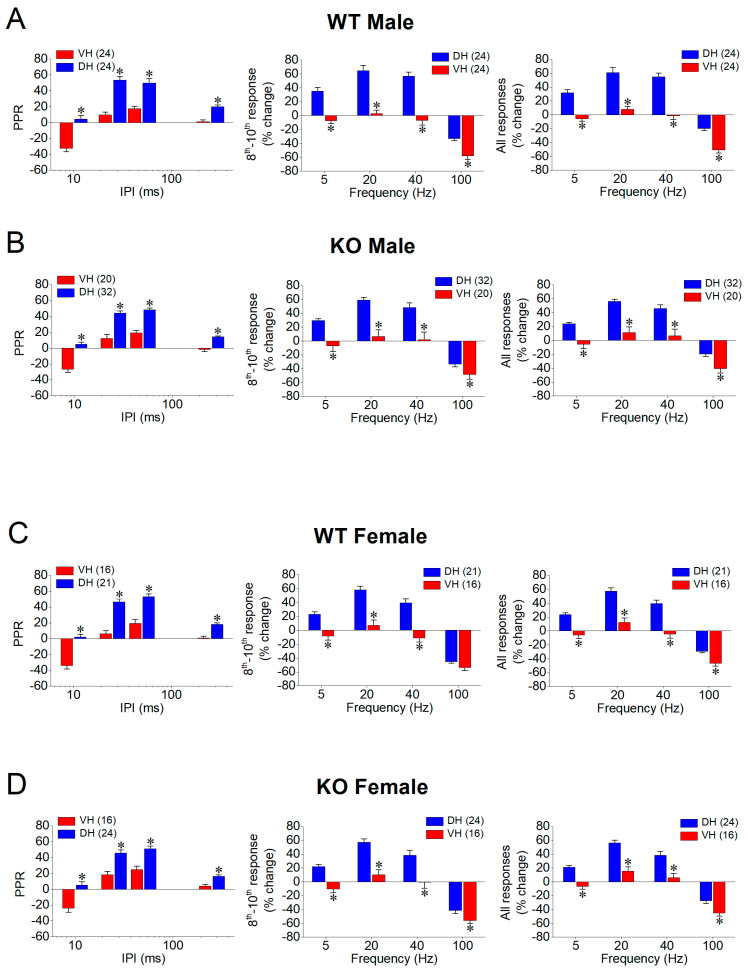
Short-term synaptic plasticity (STSP) strongly differs between dorsal (DH) and ventral (VH) hippocampus. Results are shown for males (**A**,**B**) and females (**C**,**D**), as well as for wild-type (WT) (**A**,**C**) and *Fmr1*-knockout (KO) (**B**,**D**) rats. In each of four panels (**A**–**D**) are shown average paired-pulse ratio (PPR, left), percentage of steady-state response (average of 8th–10th responses), and percentage of all conditioned responses (average of 2th–10th responses). Data presented here are replotted from Figure 1 and Figure 2 to illustrate dorsoventral differences in STSP. Numbers in parentheses indicate number of hippocampal slices used in analysis. Asterisks (*) denote statistically significant difference between DH and VH at *p* < 0.05 (independent *t*-test).

**Figure 4:**
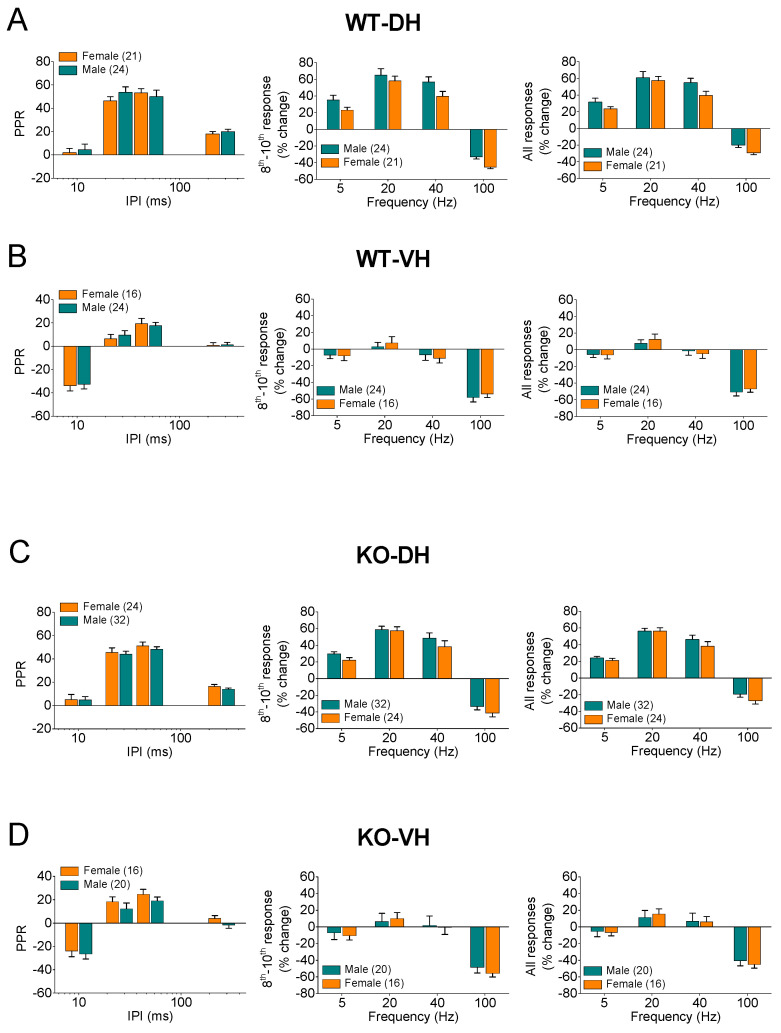
Short-term synaptic plasticity (STSP) is similar in males and females. Results are shown for wild-type (WT) (**A**,**B**) and *Fmr1*-knockout (KO) (**C**,**D**) rats, as well as for dorsal hippocampus (DH) (**A**,**C**) and ventral hippocampus (VH) (**B**,**D**) In each of four panels (**A**–**D**) are shown average paired-pulse ratio (PPR, left), percentage of steady-state response (average of 8th–10th responses), and percentage of all conditioned responses (average of 2th–10th responses). Data presented here are replotted from Figure 1 and Figure 2 to illustrate similarity in STSP between two sexes. Numbers in parentheses indicate number of hippocampal slices used in analysis.

Then, we compared the PPR and FF/D between the two segments of the hippocampus (Figure 3), as well as between the males and females (Figure 4). In the WT male rats, we confirmed previous observations showing that the PPR and FF/D differ remarkably between the DH and VH [59,60,61,62,63,64,65,66,67,68,69,70,71] (Figure 3A). Notably, the DH compared to the VH in the WT male rats showed a significantly higher PPR (DH = 24 and VH = 24, *F*_1,179_ = 65.5, *p* < 0.001) and FF/D (DH = 24 and VH = 24, *F*_1,179_ = 57.3, *p* < 0.001; and *F*_1,179_ = 65.2, *p* < 0.001; for the steady-state responses and all conditioned responses, respectively). Furthermore, we demonstrated that the remarkable dorsoventral differences were also maintained in the hippocampus of the KO male rats for the PPR (DH = 20 and VH = 32, *F*_1,200_ = 64.0, *p* < 0.001) and FF/D (DH = 20 and VH = 32, *F*_1,200_ = 34.4, *p* < 0.001; and *F*_1,200_ = 39.7, *p* < 0.001; for the steady-state responses and all conditioned responses, respectively) (Figure 3B). In addition, we report, for the first time, that similar robust dorsoventral differences also exist in the female hippocampus, both in the WT (Figure 3C) and KO (Figure 3D) rats. Specifically, the DH compared to the VH of the WT female rats show a significantly higher PPR (DH = 16 and VH = 21, *F*_1,131_ = 61.4, *p* < 0.001) and FF/D (DH = 16 and VH = 21, *F*_1,131_ = 31.4, *p* < 0.001; and *F*_1,131_ = 39.4, *p* < 0.001; for the steady-state responses and all conditioned responses, respectively). Similarly, the DH compared to the VH of the KO female rats show a significantly higher PPR (DH = 16 and VH = 24, *F*_1,150_ = 30.2, *p* < 0.001) and FF/D (DH = 16 and VH = 24, *F*_1,150_ = 22.2, *p* < 0.001; and *F*_1,150_ = 24.6, *p* < 0.001; for the steady-state responses and all conditioned responses, respectively).

Also, we found no significant gender-related differences in the STSP, in either the WT (Figure 4A,B) or KO (Figure 4C,D) rats, indicating that the robust dorsoventral differences in the STSP exist in the hippocampus of both genders. Specifically, a comparison of the WT females and males showed a similar PPR (female = 21 and male = 24, *F*_1,131_ = 0.13, *p* > 0.05) and FF/D (female = 21 and male = 24, *F*_1,164_ = 2.28, *p* > 0.05; and *F*_1,164_ = 1.81, *p* > 0.05; for the steady-state responses and all conditioned responses, respectively) in the DH, and a similar PPR (female = 16 and male = 24, *F*_1,154_ = 0.4, *p* > 0.05) and FF/D (female = 16 and male = 24, *F*_1,154_ = 0.05, *p* > 0.05; and *F*_1,131_ = 0.03, *p* > 0.05; for the steady-state responses and all conditioned responses, respectively) in the VH. Likewise, no sex-related differences were found in the KO-DH for the PPR (female = 24 and male = 32, *F*_1,217_ = 0.38, *p* > 0.05) and FF/D (female = 21 and male = 24, *F*_1,217_ = 1.1, *p* > 0.05; and *F*_1,131_ = 0.7, *p* > 0.05; for the steady-state responses and all conditioned responses, respectively), or in the KO-VH for the PPR (female = 16 and male = 20, *F*_1,133_ = 2.1, *p* > 0.05) and FF/D (female = 16 and male = 20, *F*_1,133_ = 0.02, *p* > 0.05; and *F*_1,131_ = 3.02, *p* > 0.05; for the steady-state responses and all conditioned responses, respectively).

### 2.3. Expression of Synaptotagmin-1 in WT and KO Rat Hippocampus

**Figure 5:**
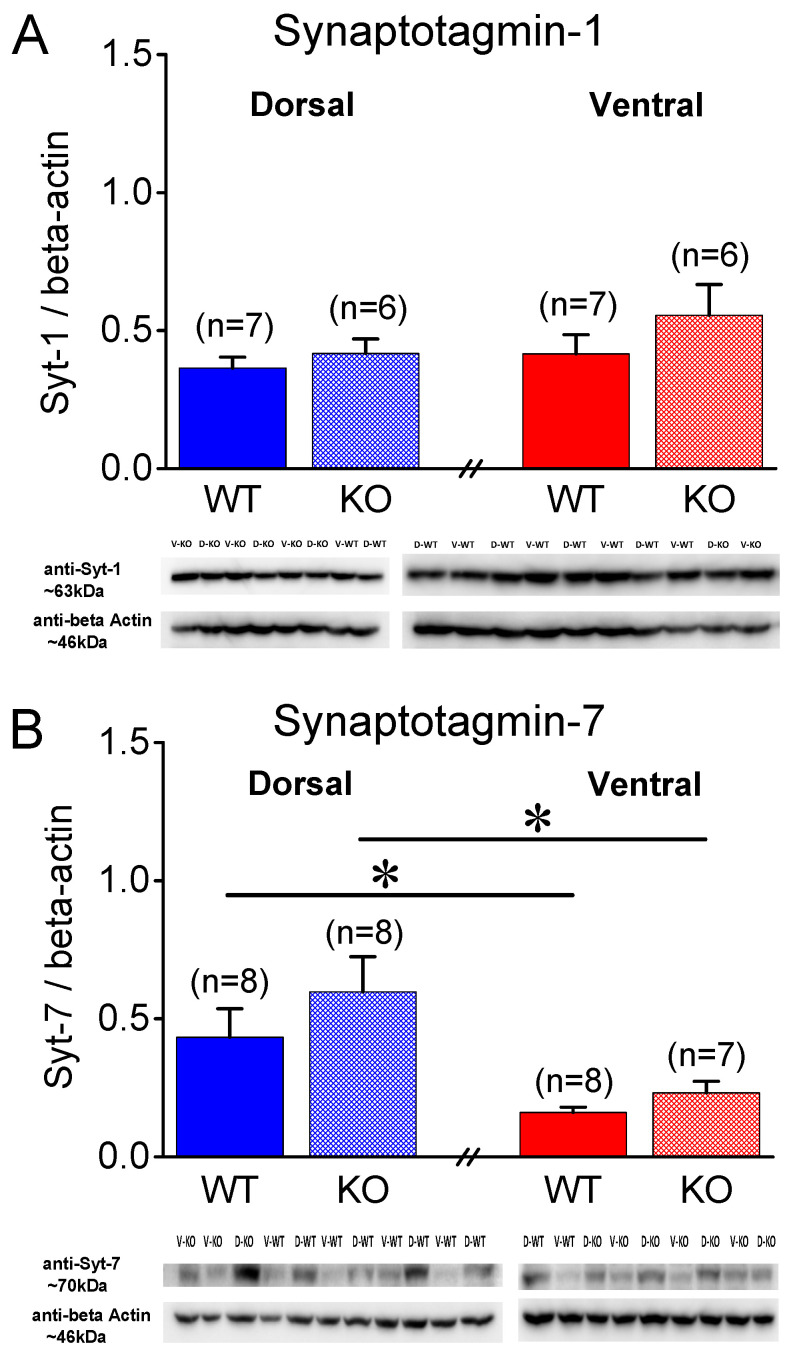
Expression of synaptotagmin-1 (Syt-1) and synaptotagmin-7 (Syt-7) in the CA1 hippocampal region of male rats. Syt-7, but not Syt-1, levels differ between dorsal and ventral hippocampus, but not between wild-type (WT) and *Fmr1*-knockout (KO) male rats. (**A**) Protein expression of Syt-1 is similar between WT and KO and between DH and VH male rats. (**B**) Protein expression of Syt-7 significantly differs between DH and VH for both genotypes, but does not differ between either segment of hippocampus for both genotypes. Numbers in parentheses indicate number of rats used in analysis. Asterisks (*) denote statistically significant differences at *p* < 0.05 (independent *t*-test).

**Figure 6:**
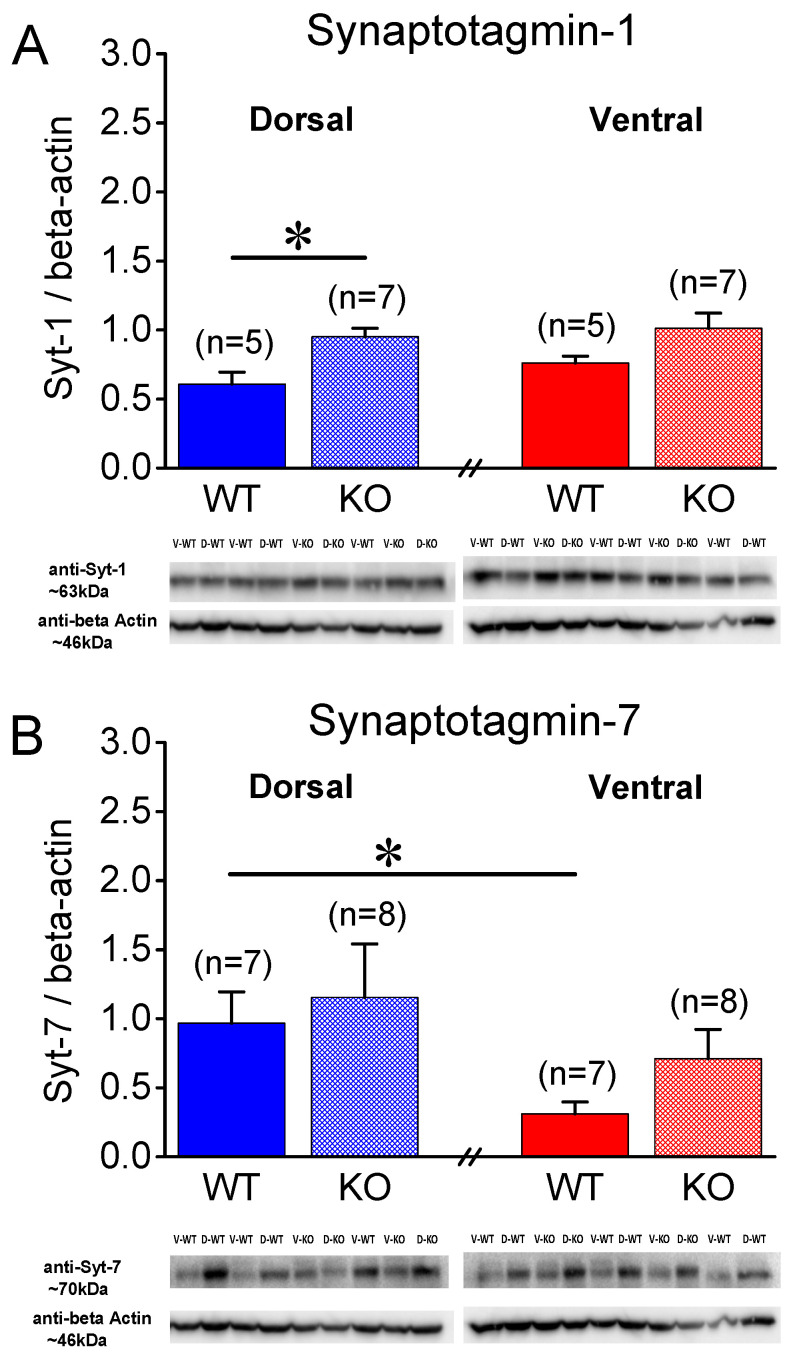
Expression of synaptotagmin-1 (Syt-1) and synaptotagmin-7 (Syt-7) in CA1 hippocampal region of female rats. (**A**) Syt-1 exhibits a higher expression in *Fmr1*-knockout (KO) than wild- type (WT) dorsal, but not ventral, hippocampus of female rats. (**B**) Protein expression levels of Syt-7 are significantly lower in VH than DH WT, but not in KO, female rats. Furthermore, Syt-7 is similarly expressed in two genotypes. Numbers in parentheses indicate number of rats used in analysis. Asterisks (*) denote statistically significant differences at *p* < 0.05 (independent *t*-test).

Syt-1 is a presynaptic protein crucially involved in synchronous neurotransmitter release [42,43]. Figure 5A shows that in male rats, Syt-1 is similarly expressed in the WT and KO DH (t_11_ = −0.821, *p* = 0.429, WT = 7 and KO = 6) and VH (t_11_ = −1.101, *p* = 0.295, WT = 7 and KO = 6). Furthermore, we found similar levels of Syt-1 in the DH and VH of both the WT (t_12_ = −0.648, *p* = 0.529) and KO male rats (t_10_ = −1.119, *p* = 0.289). In contrast to the male rats, we detected significant differences in the levels of Syt-1 in the female hippocampus. Specifically, Syt-1 was expressed in higher levels in the KO than the WT DH (t_10_ = −3.32, *p* = 0.008, WT = 5 and KO = 7), but not the VH (t_9_ = −1.941, *p* = 0.084, WT = 5 and KO = 7) (Figure 6A) of the female rats. Furthermore, we found similar levels of Syt-1 in the DH and VH of both the WT (t_8_ = −1.52, *p* = 0.168) and KO female rats (t_11_ = −0.507, *p* = 0.622).

### 2.4. Higher Levels of Synaptotagmin-7 in DH vs. VH

Then, motivated by the recently proposed positive relationship between synaptic facilitation and the expression of Syt-7 [48], we measured the levels of Syt-7 in the hippocampus of male WT and KO rats. We found that Syt-7 was similarly expressed in the WT and KO DH (t_14_ = −1.00, *p* = 0.333, WT = 8 and KO = 8) and VH (t_8.6_ = −1.523, *p* = 0.163, WT = 8 and KO = 7) of male rats. Remarkably, we found a significantly lower Syt-7 protein expression in the VH compared to the DH in both the WT (t_14_ = 2.586, *p* = 0.022) and KO (t_8.49_ = 2.72, *p* = 0.025) male rats (Figure 5B).

Regarding the female rats, we found no significant difference between the WT and KO DH (t_13_ = −0.401, *p* = 0.695, WT = 7 and KO = 8) and VH (t_9.17_ = −1.755, *p* = 0.112, WT = 7 and KO = 8). However, we found significantly lower levels of Syt-7 in the VH compared to the DH in the WT (t_7.64_ = 2.7, *p* = 0.028, DH = 7 and VH = 7), but not the KO (t_14_ = 1.0, *p* = 0.332, DH = 8 and VH = 8), female rats (Figure 6B). These results suggest that the lower levels of Syt-7 in the VH vs. DH conforms with the lower synaptic facilitation seen in the VH vs. DH in both sexes, at least in WT rats.

### 2.5. Similar Expression of AMPA Receptor Subunits GluA1 and GluA2 in WT and KO Rats

**Figure 7:**
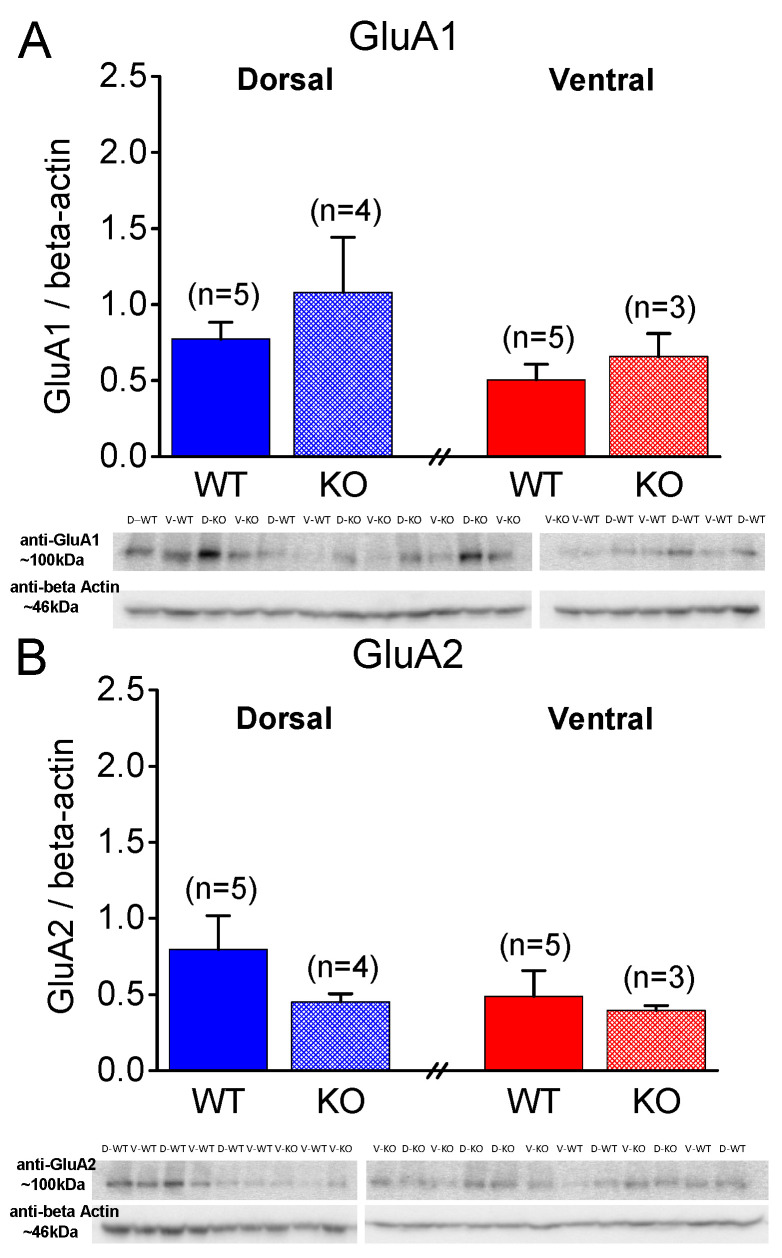
Expression of AMPA receptor subunits GluA1 and GluA2 in CA1 region of male rats. (**A**) GluA1 subunit expression is shown for wild-type (WT) and knockout (KO) dorsal hippocampus and ventral hippocampus of male rats. (**B**) GluA2 subunit expression is shown for the WT and KO DH and VH of male rats. Numbers in parentheses indicate the number of rats used in analysis.

**Figure 8:**
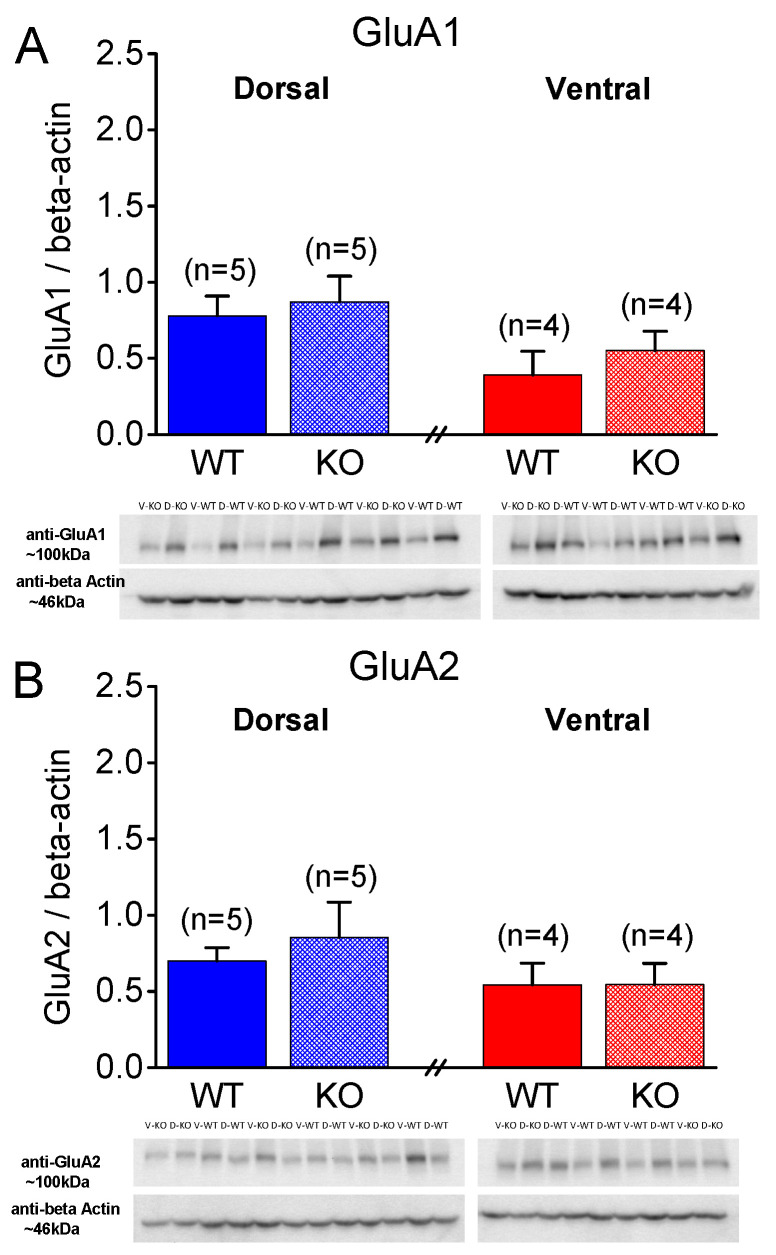
Expression of AMPA receptor subunits GluA1 and GluA2 in CA1 hippocampal region of female rats. (**A**) GluA1 subunit expression is shown for wild-type (WT) and knockout (KO) dorsal hippocampus (DH) and ventral hippocampus (VH) of male rats. (**B**) GluA2 subunit expression is shown for WT and KO DH and VH of male rats. Numbers in parentheses indicate number of rats used in analysis.

Next, considering that, in addition to presynaptic mechanisms, postsynaptic mechanisms, including AMPA receptors, can be involved in shaping the properties of STSP [78], we aimed to examine the levels of AMPA receptors by measuring the protein expression of AMPA receptor subunits GluA1 and GluA2 under the different conditions. As shown in Figure 7 and Figure 8, the protein expression of the GluA1 and GluA2 subunits in the isolated CA1 region does not significantly differ between the WT and KO dorsal or ventral hippocampus either in the males (Figure 7) or females (Figure 8). Specifically, the GluA1 subunit is similarly expressed in the WT and KO dorsal (t_3.56_ = −0.818, *p* = 0.465, WT = 5 and KO = 4) and ventral hippocampus (t_6_ = −0.878, *p* = 0.414, WT = 5 and KO = 3) of the male rats. Also, the expression of GluA1 is similar in the DH and VH of the WT male rats (t_8_ = 1.78, *p* = 0.113), and in the DH and VH of the KO male rats (t_3.95_ = 1.081, *p* = 0.341). The GluA2 subunit is similarly expressed in the WT and KO dorsal (t_4.43_ = 1.503, *p* = 0.2, WT = 5 and KO = 4) and ventral hippocampus (t_6_ = 0.41, *p* = 0.696, WT = 5 and KO = 3) of the male rats. Also, the expression of GluA2 is similar in the DH and VH of the WT male rats (t_8_ = 1.101, *p* = 0.303), and in the DH and VH of the KO male rats (t_5_ = 0.841, *p* = 0.439). Furthermore, the GluA1 subunit is similarly expressed in the WT and KO dorsal (t_8_ = −0.431, *p* = 0.678, WT = 5 and KO = 5) and ventral hippocampus (t_6_ = −0.812, *p* = 0.448, WT = 4 and KO = 4) of the female rats. Also, the expression of GluA1 is similar in the DH and VH of the WT female rats (t_7_ = 1.923, *p* = 0.096), and in the DH and VH of the KO female rats (t_7_ = 1.434, *p* = 0.195). The GluA2 subunit is similarly expressed in the WT and KO dorsal (t_8_ = −0.626, *p* = 0.549, WT = 5 and KO = 5) and ventral hippocampus (t_6_ = −0.003, *p* = 0.998, WT = 4 and KO = 4) of the female rats. Also, the expression of GluA2 is similar in the DH and VH of the WT female rats (t_7_ = 0.977, *p* = 0.361), and in the DH and VH of the KO female rats (t_7_ = 1.075, *p* = 0.318).

In addition, the GluA1 and GluA2 subunit expression in the two segments of the hippocampus were similar in the males and females with either genotype. Notably, the GluA1 subunit was similarly expressed in the male and female dorsal (t_8_ = −0.032, *p* = 0.97, male = 5 and female = 5) and ventral hippocampus (t_7_ = 0.637, *p* = 0.544, male = 5 and female = 4) of the WT rats, and in the male and female dorsal (t_4.32_ = 0.528, *p* = 0.624, male = 4 and female = 5) and ventral hippocampus (t_5_ = 0.549, *p* = 0.607, male = 3 and female = 4) of the KO rats. Likewise, the expression of the GluA2 subunit did not significantly differ between the male and female dorsal (t_5.24_ = 0.404, *p* = 0.702, male = 5 and female = 5) and ventral hippocampus (t_7_ = −0.240, *p* = 0.817, male = 5 and female = 4) of the WT rats, or between the male and female dorsal (t_7_ = −1.512, *p* = 0.174, male = 4 and female = 5) and ventral hippocampus (t_5_ = −0.888, *p* = 0.415, male = 3 and female = 4) of the KO rats.

### 2.6. Similar Excitatory Synaptic Transmission in DH and VH of WT and KO Male Rats

**Figure 9:**
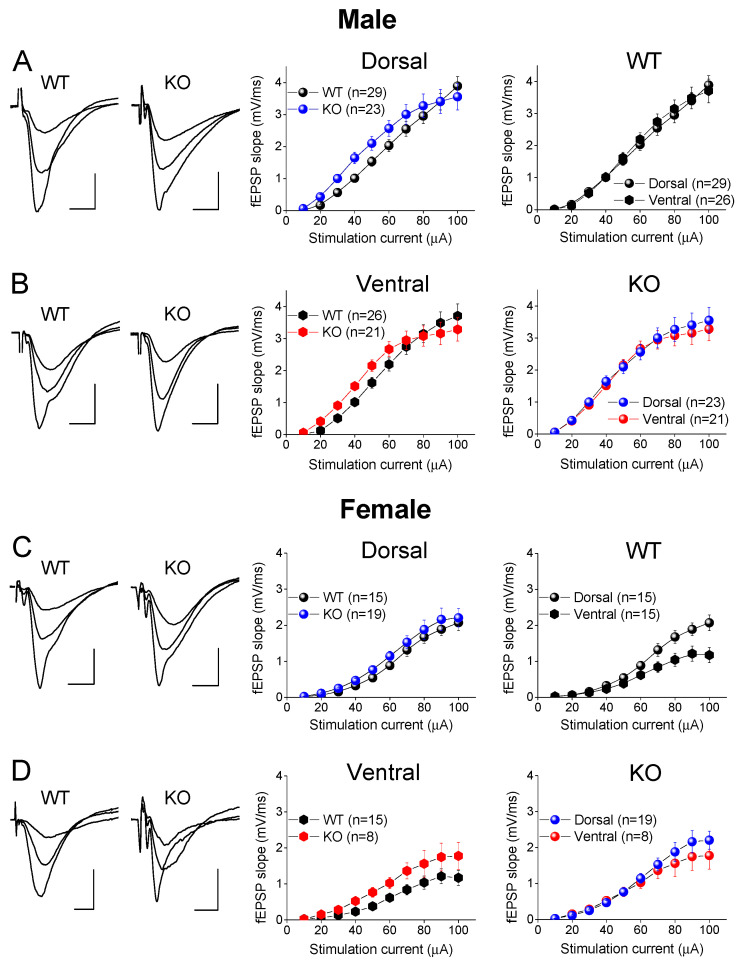
FXS does not affect excitatory synaptic transmission in either male or female dorsal and ventral hippocampus. Sample traces of synaptic responses evoked with increasing stimulation current intensity (left panels) and input–output functions between stimulation current and fEPSP (middle and right panels) obtained from dorsal and ventral hippocampus of male (**A**,**B**) and female (**C**,**D**) wild-type (WT) and *Fmr1*-knockout (KO) rats. Graphs in middle panels show input–output curves obtained from either dorsal or ventral hippocampus, while same data are replotted in right panel graphs to compare input–output relationship between dorsal and ventral hippocampus. Numbers in parentheses indicate number of hippocampal slices used in analysis.

We also examined the possible effect of genotype on basal excitatory synaptic transmission in the DH and VH of the male and female rats by constructing input–output (I-O) curves between the stimulation current intensity and fEPSP (Figure 9). We found no significant effect of genotype on the I-O relationship in the male DH (*F*_9,511_ = 0.920, *p* = 0.507; WT = 29, KO = 23; Figure 9A) and VH (*F*_9,465_ = 1.122, *p* = 0.346; WT = 26, KO = 21; Figure 9B). Similarly, the I-O curves were similar for the WT and KO female DH (*F*_9,326_ = 0.034, *p* = 0.999; WT = 15, KO = 19; Figure 9C) and VH (*F*_9,214_ = 0.639, *p* = 0.763; WT = 15, KO = 8; Figure 9D). Regarding the male rats, the present results conform with those of a previous study [73]. However, another study reported increased basal excitatory synaptic transmission in the DH of KO vs. WT male rats [72]. This inconsistency apparently results from the different measurement approach used in the two studies to estimate the excitatory synaptic transmission; in the first study, the whole I-O curve, as well as the average fEPSP calculated from the whole curve, were compared for the two genotypes, while in the second study, the average fEPSPs evoked with a moderate stimulation current intensity were measured. The data from the present study suggest that the genotype does not significantly affect basal excitatory synaptic transmission in either segment of the hippocampus in male or female rats. Furthermore, the electrophysiological results conform with the similar levels of Syt-1 for the two genotypes, at least in the males.

### 2.7. Increased Excitatory Synaptic Transmission in DH vs. VH of WT Female Rats

We also examined the effect of the hippocampal segment on basal synaptic transmission by comparing the I-O relationships between the DH and VH (see right panels in Figure 9). We found no significant dorsoventral difference in the fEPSP/I curves in either the WT male rats (*F*_9,541_ = 0.179, *p* = 0.996; DH = 29, VH = 26; Figure 9A) or the KO male rats (*F*_9,435_ = 0.122, *p* = 0.999; DH = 23, VH = 21; Figure 9B). In contrast to the male rats, we found increased excitatory synaptic transmission in the DH compared to the VH of the WT female rats (*F*_9,289_ = 2.837, *p* = 0.003; DH = 15, VH = 15; Figure 9C), but not for the KO female rats (*F*_9,251_ = 0.376, *p* = 0.946; DH = 19, VH = 8; Figure 9D). The results regarding male WT rats confirm previously reported observations [61,72,79,80,81]. Regarding female rats, to the best of our knowledge, this is the first time that basal excitatory synaptic transmission has been compared between the DH and VH in female rats.

### 2.8. Higher Excitatory Synaptic Transmission in Male vs. Female Hippocampus

**Figure 10:**
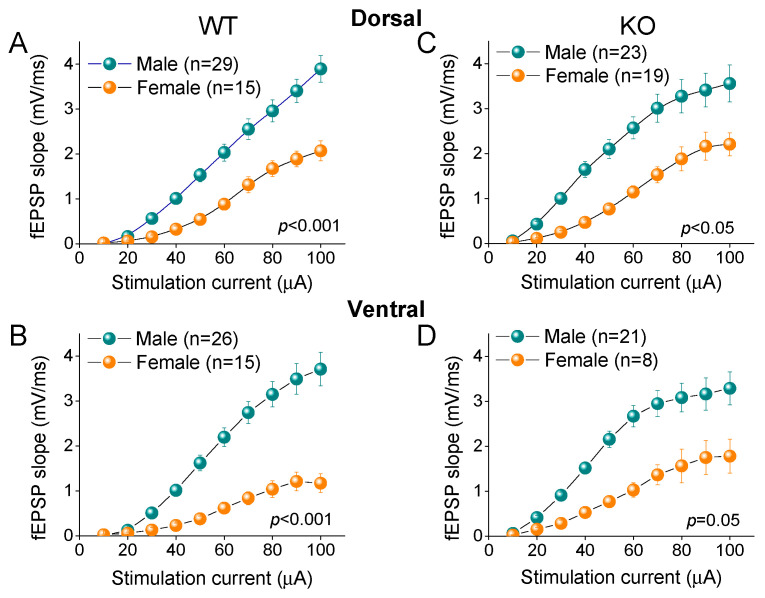
Excitatory synaptic transmission differs between males and females in both dorsal (DH) and ventral (VH) hippocampus, in either wild-type (WT) or knockout (KO) rats. Data presented here are replotted from Figure 7 to illustrate gender-associated differences in excitatory synaptic transmission (**A**–**D**). Results of two-way ANOVA used to compare I-O curves are provided in each graph. Numbers in parentheses indicate number of hippocampal slices used in analysis.

Comparing the basal excitatory synaptic transmission between the male and female rats, we found that the fEPSP was significantly higher in the male than female WT rats, both in the DH (*F*_9,433_ = 4.39, *p* < 0.001; male = 29, female = 15; Figure 10A) and VH (*F*_9,397_ = 8.14, *p* < 0.001; male = 26, female = 15; Figure 10B). Similarly, the fEPSP was significantly higher in the male than female KO rats in the DH (*F*_9,404_ = 2.0, *p* = 0.039; male = 23, female = 19; Figure 10C) and VH (*F*_9,282_ = 1.86, *p* = 0.50; male = 21, female = 8; Figure 10D). Our observations concerning the gender-dependent effects on the basal fEPSP in WT rats confirm the results of a previous study conducted in adult Long–Evans rats, which suggested that the increased basal excitatory synaptic transmission in males is most likely due to the excitatory effect of testosterone [82]. However, the effect of sex on basal synaptic transmission may depend on the strain of animals used, since another study reported greater fEPSPs in female than in male Wistar rats [83]. In addition, we show that the effect of sex on basal transmission is maintained in the KO rats, suggesting that the gender-related difference in basal excitatory synaptic transmission is unaffected by FXS.

## 3. Discussion

A key molecular signature of FXS is the loss of FMRP, a protein which plays profound roles in synaptic plasticity. It has been suggested that FMRP modulates the presynaptic proteins that are involved in neurotransmitter release and short-term synaptic plasticity (STSP), including synaptotagmins. Recent evidence has suggested that synaptotagmin-7 (Syt-7), a calcium sensor, plays a crucial role in synaptic facilitation, a ubiquitous form of STSP. Although some forms of STSP have been found altered in immature animals with FXS, whether the loss of FMRP affects STSP in adult animals remains elusive.

In the present study, we investigated two forms of STSP, namely the paired-pulse ratio (PPR) and frequency facilitation/depression (FF/D), and the expression of Syt-1, Syt-7, and AMPA receptors in the hippocampus of WT and *Fmr1*-KO rats. Notably, we prepared slices from two segments of the hippocampus, the dorsal and the ventral, and from the two sexes, male and female. The main findings of this study are the following: (a) The STSP remains normal in both segments of the hippocampus of the male rats, and in the DH of the female KO rats, but it is altered in the VH of the female KO rats; (b) the STSP greatly differs between the DH and VH, both in the males and females; (c) the large difference in the STSP between the DH and VH is paralleled by a corresponding difference in the expression of Syt-7, both in the males and females of either genotype, except for the VH of the female KO rats; (d) basal excitatory synaptic transmission is higher in the male than female hippocampus for both genotypes; (e) AMPA receptors are similarly expressed in the WT and KO, and no gender- or region-related differences were detected in receptor levels.

### 3.1. Effects of FXS on STSP and Synaptotagmins

In the present study, we found that neither the PPR nor FF/D were affected in the dorsal or ventral hippocampus of the male KO rats. Our results for the PPR are in keeping with previous studies reporting on normal paired-pulse facilitation in the hippocampus of FXS rats [15,24,56,57]. However, regarding frequency facilitation, the present findings do not fit well with the results of previous reports, which have shown increased responses at stimulation frequencies of 20–100 Hz [15,24,57], or reduced responses at 10–20 Hz [17]. This inconsistency could be due to some important methodological differences. Notably, mouse models of FXS were used in previous studies, while we used a rat model of FXS. However, most important for this discrepancy is the age difference among the animals used in the present and previous studies. The previous studies were performed on immature animals aged between two and three weeks [15,17,24,56,57] and six weeks, while we used adult animals. FXS is a developmental disorder, and age matters for both neurobiological properties [84,85,86] and phenotypic traits [1]. For instance, the seizures that are a common trait of FXS in young individuals [2,87], often involving the anterior hippocampus (which corresponds to the ventral hippocampus in rodents), are absent in adults [2,87,88]. Moreover, the recently shown increase in GABAergic inhibition, specifically in the ventral hippocampus of adult KO rats [72,73], the brain region most susceptible to epilepsy [89,90], may be the result of homeostatic mechanisms activated during the development of individuals with FXS, in an attempt to compensate for the increased excitability of the brain. Thus, it is tempting to speculate that the normal STSP we found in the adult male KO rats may be the result of adaptive mechanisms attempting to restore the disrupted STSP in immature KO animals [15,17,24,57]. The data from a recent study using the valproic acid animal model of autism [91] are suggestive of the existence of such compensatory mechanisms during development.

Most of the previous studies that have examined STSP in the hippocampus of FXS animals were performed on slices from the middle rather than the dorsal or the ventral hippocampus [15,17,24,57]; in one study, no information was provided regarding the segment of the hippocampus used [56]. Furthermore, previous studies have not clearly distinguished between males and females. Τhe present study is the first that examines the effects of FXS on hippocampal STSP in the two genders separately, and it reveals that FXS is associated with an increase in the PPR, specifically in the ventral hippocampus of adult female KO rats. To the best of our knowledge, there are no previously published data on STSP in female FXS animals. Given the multiple roles of FMRP in synapses [7,12,13,14,15,16,17,18], these changes could possibly be related to the significant synaptic reorganization that takes place in the hippocampus of *Fmr1*-KO animals [17].

Curiously, frequency facilitation at stimulation frequencies around 40 Hz has been previously observed in studies where both males and females were used [15,57]. Interestingly, the increase in synaptic facilitation seen in the VH of the KO female rats occurred at an IPI of 25 ms, which corresponds to 40 Hz, the typical frequency of gamma oscillation that fundamentally supports the processing of neural information in the brain [92], including the processing of visual information, attention, and episodic memory. Thus, the alteration in the PPR observed here may be related to the previously reported impairment of sensory processing in females [93,94].

FMRP acts primarily by suppressing the translation of specific mRNAs, thereby affecting neuronal function and leading to the deficits observed in individuals with FXS [13,14]. However, not all mRNAs are FMRP targets [95]. Here, we show that neither Syt-1 nor Syt-7 are affected in adult FXS rats, except Syt-1, which exhibits an increased expression in the DH of adult female FXS rats. These results may indicate that synaptotagmins either escape from the consequences of FMRP loss, or their expression recovers during adulthood.

### 3.2. Dorsoventral Difference in STSP and the Possible Role of Synaptotagmin-7

In the present study, we show that both forms of STSP, i.e., the PPR and FF/D at CA3-CA1 synapses, greatly differ between the two segments of the hippocampus in male and female WT and KO rats. The difference in short-term forms of synaptic plasticity between the DH and VH CA3-CA1 synapses has been repeatedly documented previously [59,60,61,62,63,64,65,66,67,68,69,70,71], representing one of the most prominent features of intrinsic diversification along the hippocampus. Therefore, the present results confirm previous ones, and extend these findings to *Fmr1*-KO rats. However, even though this dorsoventral difference is well documented, the underlying mechanisms remain elusive. An early hypothesis, driven by the previously shown inverse relationship between the basal probability of neurotransmitter release and synaptic facilitation [32,96], proposed that a high basal transmitter release probability may be responsible for the remarkably reduced synaptic facilitation of CA1 synapses in the VH compared to the DH [65,66,97]. However, this hypothesis has not been confirmed by more recent evidence [62,69,98].

Generally, several forms of STSP are thought to involve Ca^2+^-dependent mechanisms at the presynaptic terminal [32]. Notably, synaptic facilitation has been proposed as arising from an accumulation of Ca^2+^ in the cytosol of the presynaptic terminal during repetitive activation, the so-called “residual Ca^2+^”, due to the slow action of the mechanisms that restore Ca^2+^ concentration to resting levels, leading to an increased probability of neurotransmitter release [32,38,99]. The arrival of a subsequent action potential during the period of residual Ca^2+^ could lead to increased transmitter release (synaptic facilitation).

The presynaptic proteins that belong to the family of synaptotagmins, which function as Ca^2+^ sensors and trigger neurotransmitter release by interacting with the core membrane fusion proteins [41,42,55], appear to play fundamental roles in the process of neurotransmitter release and the Ca^2+^-dependent facilitation of release [40,41]. Among the several members of this family, Syt-1 is a protein of the synaptic vesicle membrane that functions as the primary sensor for Ca^2+^ in the presynaptic terminal, triggering the rapid fusion of synaptic vesicles with the plasma membrane [42,43,100]. The low Ca^2+^ affinity of Syt-1 allows it to contribute decisively to the immediate transmitter release triggered by an action potential that leads to an abrupt and robust influx of Ca^2+^ in the vicinity of readily releasable synaptic vesicles [42,43,100].

Syt-7, on the other hand, exhibits high Ca^2+^ sensitivity and is activated at low Ca^2+^ concentrations, such as those occurring in the presynaptic terminal after an action potential invades the presynaptic terminal, when the Ca^2+^ concentration falls but remains above resting levels (residual Ca^2+^) [55]. Accordingly, recent data has suggested that, by virtue of its activation at low Ca^2+^ concentrations, Syt-7 can, crucially, support synaptic facilitation [48,49,50,51,52]. Thus, it might be expected that a relatively increased expression of Syt-7 in the presynaptic terminal would lead to a greater facilitation of synaptic transmission during rapidly repetitive activity. Indeed, the present study reveals a striking analogy between the dorsoventral difference in STSP and the levels of Syt-7 in the two segments of the hippocampus, in both the WT and KO male rats and in the WT female rats. It is, therefore, tempting to assume that the difference in synaptic facilitation observed between the DH and VH could, to some extent, be attributed to the conspicuously different expression of Syt-7 in the two segments. The similar expression of Syt-7 in the WT and KO rats suggests that Syt-7 remains unaffected by the loss of FMRP in adult rats. Thus, it will be interesting to see whether FXS affects the expression of Syt-7 in the hippocampus of immature WT and KO rats.

In addition to the presynaptic proteins that directly participate in neurotransmitter release and the facilitation of the release process, other proteins might play important roles in synaptic facilitation. For instance, a recent study has revealed that activin A, a member of the transforming growth factor B family (TGF-β), in addition to its roles in a variety of functions such as cell proliferation and neuroprotection [101], also contributes to shaping the frequency-dependent properties of synaptic facilitation in the DH and VH [59]; however, activin A does not appear to be a crucial factor for the large dorsoventral difference in synaptic facilitation.

### 3.3. Conclusions

The present findings show that the loss of FMRP is associated with an altered PPR in the VH of female KO rats. Furthermore, the increased synaptic facilitation seen in the dorsal vs. ventral hippocampus of both genotypes is accompanied by a corresponding increased level of Syt-7, suggesting that Syt-7 plays a crucial role in differentiating the properties of STSP along the longitudinal axis of the hippocampus.

Here, we should mention some of the limitations that accompany this study. For example, we examined only two forms of STSP and found that they are not significantly altered in male KO animals. However, STSP includes a significant variety of phenomena that depend on both the frequency and duration of activation (i.e., number of stimulation pulses), that may involve different mechanisms, potentially serve different functions, and may be affected differently in neurodevelopmental disorders. Therefore, we cannot rule out that there may be changes in other STSP phenomena. Also, the increased PPR we found in the ventral hippocampus of the KO females may possibly be underestimated, because WT females, due to their genetic diversity, can express FMRP in varying degrees. However, in the present study we did not quantitatively measure the FMRP. Also, we did not measure estrogen levels, which could affect protein expression and synaptic plasticity.

Finally, the fact that the data from different studies have shown that the effect of FXS on STSP varies during development is suggestive of the need to examine multiple developmental stages in a study. The elucidation of the developmental profile of STSP in both segments of the hippocampus would make it possible to identify the developmental stage in which the initial absence of FMRP is possibly followed by compensations that tend to maintain normal synaptic plasticity. Recent studies have suggested the possible existence of such compensatory mechanisms in the hippocampus of FXS rats. Developmental studies could constitute a future roadmap for investigating the effects of FXS on the neurophysiology of the hippocampus, contributing to the understanding of the causal relationship between loss of FMRP and STSP.

## 4. Materials and Methods

### 4.1. Animals

Littermates of *Fmr1*-knockout and wild-type Long–Evans rats were obtained from the Medical College of Wisconsin (RRIDs: RGD_2308852 and RGD_11553873, respectively). Adult 3–4-month-old rats of both sexes were used in this study. The female rats weighed 380–430 g, and the male rats weighed 600–700 g. Specific pathogen-free rats were maintained at the Laboratory of Experimental Animals at the Department of Medicine of the University of Patras (license No.: EL-13-BIOexp-04). The animals were kept under a stable cycle of light–dark (12/12 h) and at a temperature of 20–22 °C, and they had free access to food and water. The treatment of the animals and all the experimental procedures used in this study were conducted in accordance with the European Communities Council Directive Guidelines for the care and use of laboratory animals (2010/63/EU—European Commission). Also, the experimental procedures were approved by the Protocol Evaluation Committee of the Department of Medicine of the University of Patras and the Directorate of Veterinary Services of the Achaia Prefecture of Western Greece Region (reg. number: 5661/37, 18 January 2021). Furthermore, this animal study was reviewed and approved by the Research Ethics Committee of the University of Patras. The rats were genotyped after each experiment using tail or brain tissue to test for the expression of FMRP protein by means of Western blotting.

### 4.2. Slice Preparation

The slices were prepared from the dorsal and the ventral hippocampus as previously described [68]. Briefly, following the decapitation of the animal under deep anesthesia with diethyl-ether, the brain was removed from the skull, and placed in chilled (2–4 °C) artificial cerebrospinal fluid (ACSF) containing (in mM) 124 NaCl, 4 KCl, 2 CaCl_2_, 2 MgSO_4_, 26 NaHCO_3_, 1.25 NaH_2_PO_4_, and 10 glucose. The ACSF was equilibrated with a 95% O_2_ and 5% CO_2_ gas mixture at a pH = 7.4. After excising the two hippocampi from the hemispheres, we prepared 550 μm thick slices from the dorsal and ventral hippocampus, cutting transversally to the long axis of the structures using a McIlwain tissue chopper. The slices were prepared from segments of the hippocampus extending between 0.5 mm and 3.5 mm from each end. The slices were immediately transferred one by one, after preparation, into a homemade, interface-type (air–liquid) recording chamber where they were maintained for the rest of the experiment, and were continuously perfused with ACSF and humidified with a gas mixture of the same composition as described above, at a temperature of 30 ± 0.5 °C. The slices were perfused at a rate of ~1.5 mL/min and left to recover from the cut for at least one-and-a-half hours after their placement in the recording chamber.

### 4.3. Electrophysiology

Recordings of the evoked field excitatory postsynaptic potentials (fEPSPs) were performed on the stratum radiatum of the middle CA1 hippocampal region, using a 7 μm thick carbon fiber electrode (Kation Scientific, Minneapolis, MN, USA). The fEPSPs were evoked using electrical stimulation of the Schaffer collaterals by a homemade bipolar platinum/iridium wire electrode, with a wire diameter of 25 μm (World Precision Instruments, Sarasota USA), and an inter-wire distance of 100 μm. Stimulation current pulses of variable amplitude and a stable duration of 100 μs were delivered using a DS3 constant current stimulator (Digitimer Ltd., Hertfordshire, UK). Stimulation and recording electrodes were placed in the slices under visual guidance using three-axis mechanical micromanipulators (Narishige Group, Tokyo, Japan) and a stereo microscope (Olympus, Tokyo, Japan) under fiber optic lighting (Volpi AG, NY, USA). We applied baseline stimulation at a frequency of 0.033 Hz using a current stimulation intensity evoking an fEPSP with a slope of about 1 mV/ms. We measured the maximum slope of the early rising phase of the fEPSP. We assessed the synaptic effectiveness by systematically constructing input–output (I-O) curves for the increasing stimulation current intensity and fEPSP. Due to variations in the stimulus intensity between the experiments, the current intensity was normalized in each experiment (i.e., slice) to the maximum current intensity value used. We studied the STSP by using a frequency stimulation protocol as previously described [67,68]. Specifically, the frequency stimulation protocol consisted of short trains of ten pulses delivered at frequencies of 5, 20, 40 and 100 Hz. Consecutive stimulation trains were separated by two-minute-long intervals. The effect of repeated stimulation on the fEPSP was quantified by the percent change in either the second response or the 8–10th responses, with respect to the first conditioning response. The former approach represents the paired-pulse ratio (PPR), while the second approach provides a quantification of the postsynaptic response change in a steady-state. In addition, we calculated the average percentage of all the conditioned responses (i.e., 2–10th).

The electrical signal was acquired and amplified X500 and then filtered at 0.5 Hz–2 kHz using Neurolog systems (Digitimer Ltd., UK). The analog signal was digitized at 10 kHz using a CED 1401-plus interface and Signal 5.09 software (Cambridge Electronic Design, Cambridge, UK). The signal was continuously monitored visually and auditorily using an analog-to-digital oscilloscope (Hameg Instruments, Mainhausen, Germany) and a Neurolog audio amplifier, respectively. The signal was stored on a computer disk for off-line analysis.

### 4.4. Immunoblotting

The CA1 region of the dorsal and ventral hippocampus of the male and female WT and KO rats, and the remaining brain tissue or tail tissue, were stored at −80 °C for protein expression analysis. Following SDS-PAGE electrophoresis on 10% polyacrylamide gels, the proteins were transferred to a polyvinylidene difluoride (PVDF) membrane at 400 mA for 90 min, and the membranes were blocked with phosphate-buffered saline (PBS) containing 0.1% Tween-20 (PBST) and 5% nonfat dried milk, at room temperature. The membranes were next incubated overnight at 4 °C with the following primary antibodies diluted in PBST and 3% dried milk: rabbit anti-FMRP polyclonal (1:1500, #17722, Abcam, Cambridge, UK), mouse anti-synaptotagmin 1 monoclonal (1:1000, #MAB5200, Millipore Sigma, Burlington, MA, USA), mouse anti-synaptotagmin 7 monoclonal (1:350, #MA5-27654, Thermo Fisher Scientific, Waltham, MA, USA), rabbit anti-GluA1 polyclonal (1:2000, #D4N9V, Cell Signaling Technology, Danvers, MA, USA), rabbit anti-GluA2 polyclonal (1:2000, #E1L8U, Cell Signaling Technology, Danvers, MA, USA), and rabbit anti-beta-actin polyclonal (1:15,000, #E-AB-20058, Elabscience, Houston, TX, USA) antibodies. The blots were rinsed with PBST and then incubated with either goat anti-rabbit (1:20,000 #AP132P, Millipore Sigma, Burlington, MA, USA) or anti-mouse (1:25,000, A16084, Thermo Fisher Scientific, Waltham, MA, USA) secondary horseradish peroxidase-conjugated IgG antibodies for 60 min at RT. The molecular masses were determined by comparison with prestained protein molecular weight marker standards (27–200 kDa) (#SDS7B2, Millipore Sigma, St. Louis, MA, USA). The bands were visualized on a ChemiDoc MP (BioRad, California, USA) by enhanced chemiluminescence (Immobilon ECL Ultra Western HRP Substrate, # WBULS0500, Millipore Sigma, Burlington, MA, USA) for 1 to 10 min. The densitometric quantification of the immunopositive bands was carried out. The optical density measurements for each band were defined with ImageLab 6.1 software. The ROD of each band of proteins of interest and the ROD of beta-actin, which served as the gel-loading control, were quantified. Then, the ratio of (ROD of protein of interest)/(ROD beta-Actin) was normalized with the same ratio of an internal sample, which was loaded in all the gels.

### 4.5. Statistical Analysis

The values throughout this paper represent the mean ± S.E.M. For comparisons between the two different populations of data, we used an independent *t*-test, which accounts for unequal variances whenever needed. For comparisons of the data with repeated measures, we used a two-way ANOVA (UNIANOVA). Every possible comparison between the study groups was considered. We also examined the equality of variances and the normality of the distribution of the values of the various variables using Leven’s test and the Shapiro–Wilk test, respectively. The experimental unit was the slice in electrophysiology and the rat in the Western blotting. The IBM SPSS Statistics 27 software package was used for all the statistical analyses.

## Data Availability

All data associated with this study are available from the corresponding author upon reasonable request.

## Abbreviations

ACSF	artificial cerebrospinal fluid
fEPSP	field excitatory postsynaptic potential
DH	dorsal hippocampus
FF/D	frequency facilitation/depression
FXS	fragile X syndrome;
KO	*Fmr1* *knockout*
PPR	paired-pulse ratio
STSP	short-term synaptic plasticity
Syt-1	synaptotagmin-1
Syt-7	synaptotagmin-7
VH	ventral hippocampus
WT	wild type
